# Comprehensive genomic and immunohistochemical profiles and outcomes of immunotherapy in patients with recurrent or advanced cervical cancer

**DOI:** 10.3389/fonc.2023.1156973

**Published:** 2023-05-15

**Authors:** Yoo-Na Kim, Kyunglim Lee, Eunhyang Park, Junsik Park, Yong Jae Lee, Eun Ji Nam, Sang Wun Kim, Sunghoon Kim, Young Tae Kim, Jung-Yun Lee

**Affiliations:** ^1^ Department of Obstetrics and Gynecology, Institute of Women’s Life Medical Science, Yonsei University College of Medicine, Seoul, Republic of Korea; ^2^ Department of Pathology, Institute of Women’s Life Medical Science, Yonsei University College of Medicine, Seoul, Republic of Korea

**Keywords:** NGS, immunohistochemistry, immunotherapy, cervical cancer, personalized medicine

## Abstract

**Purpose:**

This study aimed to investigate genomic and immunohistochemical (IHC) profiles and immunotherapy outcomes in patients with cervical cancer.

**Methods:**

Patients with recurrent cervical cancer who underwent tumor next-generation sequencing (NGS) with the TruSight Oncology 500 panel at Yonsei Cancer Center between June 2019 and February 2022, were identified. Patients who received treatment with checkpoint inhibitors during the same period were also identified. Clinical information, including histology, stage, human papillomavirus (HPV) genotype, IHCs profile, and therapy outcome, was reviewed.

**Results:**

We identified 115 patients treated for recurrent cervical cancer, including 74 patients who underwent tumor NGS. Most of these 74 patients were initially diagnosed with advanced stage (63.6%) and had squamous cell histology (52.7%), and high-risk HPV (76.9%). Based on IHC analysis, the programmed death-ligand 1 combined positive score (PD-L1 CPS) was higher in patients with squamous cell carcinoma (SCC) than in those with adeno or mucinous types (P=0.020). HER2 receptor expression of 2+ and 3+ were identified in 5 and 1 patients, respectively, and significantly varied based on histology (p=0.002). Among the 74 patients, single nucleotide variants (SNVs) and copy number variations (CNVs) were identified in 60 (81.1%) and 13 patients (17.6%), respectively. The most common SNVs were PIK3CA, TP53, STK11, FAT1, and FBXW7 mutations. Mutations in PIK3CA, with two hotspot mutations, were frequently observed in patients with SCC histology, whereas mutations in TP53 were frequently observed in patients with non-SCC histology. Additionally, variations in FAT1 were exclusively identified in patients with SCC histology. Mutations in homologous recombination repair-associated genes were identified in 18 patients (24.3%). The most frequent CNV alteration was CCNE1 amplification. Moreover, among the 36 patients who underwent NGS and received immunotherapy, the tumor mutational burden and microsatellite instability were significantly correlated with immunotherapy duration. During this timeframe, 73 patients received pembrolizumab monotherapy, among whom a small portion showed a durable response.

**Conclusion:**

Comprehensive genomic and IHC profiling may help identify potential candidates for targeted immunotherapy in patients with cervical cancer.

## 1 Introduction

Cervical cancer, frequently caused by infections with the human papillomavirus (HPV), is the fourth most common cancer among women globally, with an estimated 604,000 cases and 342,000 deaths reported in 2020 (1). Owing to vaccination against HP, early screening, and early intervention with conization, early-stage cervical cancer is effectively treated and controlled. However, the prognosis of advanced-stage cervical cancer remains poor, with a 5-year survival rate of 39%, 24%,15%, and less than 5% for stage III, stage Iva, stage IVb, and recurrent cancer, respectively (2, 3). Despite various preventive and early intervention strategies, the mortality rate of cervical cancer has not improved, suggesting that the standard treatment with platinum-based chemotherapy is insufficient for advanced-stage cervical cancer.

Various non-chemotherapeutic options, such as immune checkpoint inhibitors (ICI) and targeted agents, have been investigated to improve survival outcomes in cervical cancer (4–8). Of these, pembrolizumab, a programmed death-1 (PD-1) receptor inhibitor, is widely investigated and has received US Food and Drug Administration approval for patients with persistent, recurrent, or metastatic cervical cancer with a PD-L1 combined positive score (CPS) of ≥1 based on Keynote-158 (9). Previously reported predictive biomarkers for ICI include PD-L1 immunohistochemistry (IHC) and genomic assays, such as tumor mutational burden (TMB) or microsatellite instability (MSI) (10). Owing to the increased clinical use of next-generation sequencing (NGS), mutational profiling may help in further personalizing therapy for cervical cancer. Moreover, previous studies on the genomic landscape of cervical cancer have identified frequent alterations in genes, such as PIK3CA, EP300, FBXW7, and APOBEC signatures, associated with the process of carcinogenesis of virus-associated diseases (11, 12).

This study aimed to present a comprehensive profile of IHC and genomic biomarkers in patients with cervical cancer and the outcomes of immunotherapy from a single institution. We identified patients with cervical cancer who underwent tumor NGS with the TruSight Oncology 500 panel and collected clinical parameters such as histology, HPV genotyping, tumor markers, and IHC results for PD-L1 and human epidermal growth factor receptor 2 (HER2) receptor status. Furthermore, we investigated all patients with cervical cancer who received pembrolizumab monotherapy within the same timeframe to provide real-world data on immunotherapy outcomes.

## 2 Materials and methods

### 2.1 Patient recruitment and sample acquisition

Patients who were diagnosed with cervical cancer between June 2019 and February 2022 at Yonsei Cancer Hospital and underwent NGS with TruSight Oncology 500 were retrospectively identified. During the same period, all patients with cervical cancer who received pembrolizumab monotherapy were also identified. This study was approved by the hospital’s institutional review board (IRB No # 4-2022-1399). The need for informed consent was waived because of the retrospective nature of the study.

### 2.2 NGS of tumor samples

Tumor samples were prepared from formalin-fixed paraffin-embedded (FFPE) tissues. An expert pathologist reviewed the hematoxylin and eosin-stained slides to ensure adequate tumor content. For DNA extraction, 2–5 slides of resected specimens with a thickness of 5 µm were used. FFPE samples with high tumor cellularity (>10%) were subjected to NGS analysis. Genomic DNA was extracted using a Maxwell CSC DNA FFPE Kit (Promega, Madison, WI, USA), according to the manufacturer’s instructions. The products were sequenced using the NextSeq 550 System (Illumina Inc., San Diego, CA, USA). Mutational and copy number analyses were performed using a TruSight Oncology 500 panel (Illumina). For mutational analysis, FASTQ files were uploaded to the Illumina BaseSpace software (Illumina) for variant interpretation. Only variants in coding regions, promoter regions, or splice variants were retained. In addition, we only retained variants present in 3% of the reads, with a minimum read depth of 250. All retained variants were reviewed against reference websites (Catalogue of Somatic Mutations in Cancer [http://evs.gs.washington.edu/EVS/], Precision Oncology Knowledge Base [http://oncokb.org], and dbSNP [https://www.ncbi.nlm.nih.gov/snp]). Only pathogenic variants were selected for further analysis. In the copy number analysis, only genes with more than a two-fold change relative to the average level were considered for amplification. TMB and MSI scores were obtained for patients who underwent NGS using the TruSight Oncology 500 panel.

### 2.3 IHC

FFPE tissue specimens were used for IHC analysis. After deparaffinization with xylene and rehydration with an alcohol-graded solution, IHC was performed using a Ventana Discovery XT Automated Slide Stainer (Ventana Medical System, Oro Valley, AZ, USA). Cell conditioning 1 buffer (citrate buffer, pH 6.0; Ventana Medical System) was used for antigen retrieval. Sections were incubated with primary antibodies against PD-L1 (1:50; clone 22C3; DAKO, Agilent Technologies, Santa Clara, CA, USA) and HER2 (1:1500; polyclonal; DAKO). For PD-L1 expression, CPS (1:50; clone 22C3; DAKO) was calculated as previously described (13). HER2 IHC was assessed according to the American Society of Clinical Oncology/College of American Pathologists guidelines based on the grading system, ranging from 0 to 3+ (14).

### 2.4 Collection of information on clinical variables, treatment received, and outcomes

Basic clinical information, such as age at diagnosis, histology, serum tumor markers, and FIGO stage at diagnosis, was obtained. We also assessed whether the patients received ICI or targeted therapy, and the name of the therapeutic agent, treatment duration, and date of disease progression were recorded.

### 2.5 Statistical analysis

Statistical analysis was performed using R version 4.0.3 (R Foundation for Statistical Computing, Vienna, Austria). Variant calling file from the aforementioned NGS pipeline was used for analysis and visualization using the “maftools” package in R.

Significance was calculated using Fisher’s exact test or chi-square test for categorical variables and Student’s *t*-test for continuous variables, where applicable. The Kaplan–Meier method was used to analyze treatment response and overall survival. For all analyses, significance was set at P < 0.05.

## 3 Results

A total of 115 patients with cervical cancer who either underwent NGS or received pembrolizumab were identified. Among these patients, the clinical characteristics and IHC profiles of 74 patients with NGS data were analyzed (**Supplementary Table 1**). These patients had advanced-stage cervical cancer (63.6%), squamous cell histology (52.7%), and high-risk HPV genotype (76.9%), and 61 patients (82.4%) harbored either single nucleotide variant (SNV) or gene copy number variant (CNV) alterations (**Figure S1**). The overall landscape of the pathogenic SNV alterations is shown in **Figure S1**. The most common alterations were observed in PIK3CA and TP53. PIK3CA showed two hotspot mutations, E542K and E545K, in 19 of the 23 (82.6%) patients with PIK3CA mutations. The most frequently highlighted pathways were PI3K, TP53, and Notch (**Figure S1**). Based on the somatic interaction plot, ERBB2, STK11, PIK3R1, PTEN, ARID1A, and CREEBP mutations were found to co-occur (**Figure S1**).

**Figure 1:**
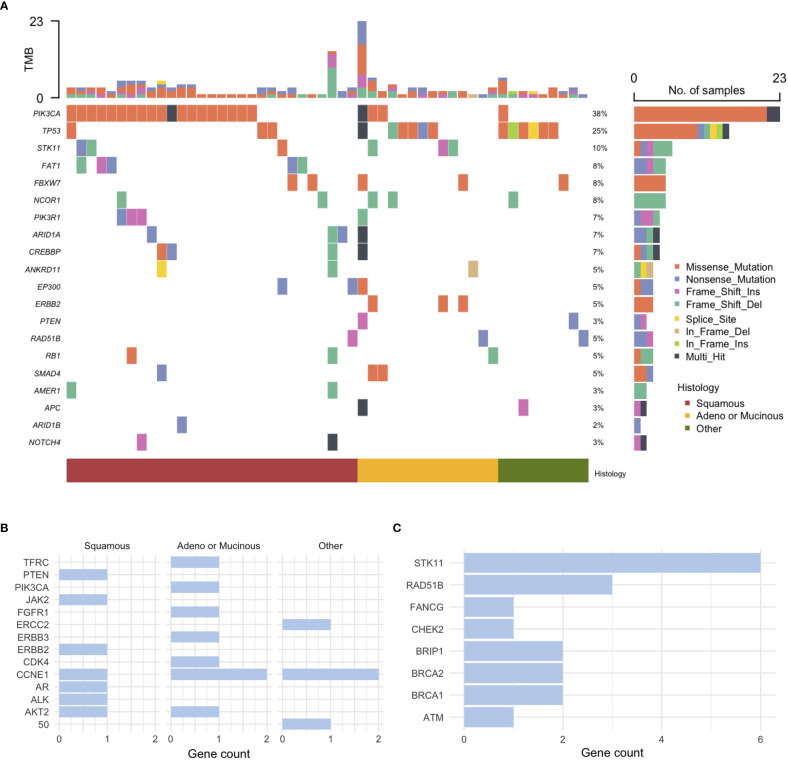
Genomic profiling of cervical cancer. **(A)** Pathogenic single nucleotide variant (SNV) alterations, **(B)** Copy number variation (CNV) alterations stratified by histology, and **(C)** mutations in homologous recombination repair-associated genes.

Pathogenic SNV alterations stratified by histology are shown in **Figure 1A**. Mutations in PIK3CA were relatively more common in patients with squamous cell histology, whereas mutations in TP53 were relatively more common in those with non-squamous histology. Mutations in FAT1 were exclusively identified in patients with squamous cell histology. The most common CNVs were CCNE1 amplification in five patients and ERBB2/3 amplification in two patients. CNV alterations based on histology are shown in **Figure 1B**. Pathogenic mutations in homologous repair (HRR)-associated genes were identified in 18 of 74 (24.3%) patients. Mutations in HRR-associated genes based on histology are shown in **Figure 1C**.

**Figure 2:**
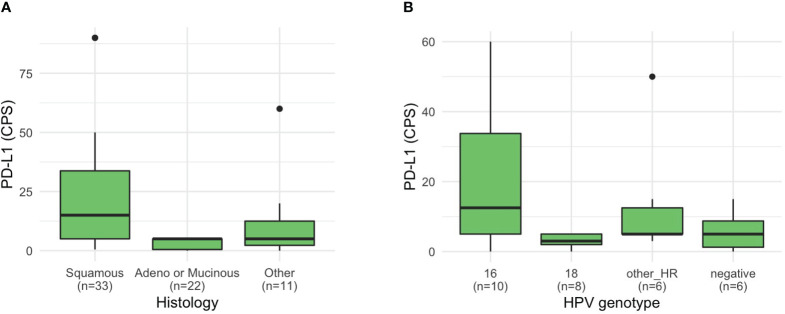
PD-L1 CPS of patients with cervical cancer. PD-L1 CPS based on **(A)** histology and **(B)** HPV genotype.

Clinical variables such as HPV genotype, serum tumor markers, and IHC were differentially distributed based on histology (**Supplementary Table 2**). Patients with squamous cell histology frequently harbored HPV 16 (46.2%) and high-risk genotype other than HPV 16 or 18 (26.9%), whereas patients with non-squamous frequently harbored HPV 18 (36.8% of patients with adeno or mucinous histology; 42.9% with other histology). Among the 45 patients who were investigated, the PD-L1 CPS score was higher in patients with squamous cell histology (median 15, range 0.5–90) than in those with adeno or mucinous histology (median 5, range 0–5) (P = 0.013; **Figure 2**). Moreover, among 39 patients tested for HER2 expression, most patients (90.9%) with squamous cell histology lacked HER2 expression (**Supplementary Table 2**). HER2 expression of 2+ or 3+ was relatively more common in patients with non-squamous histology (P = 0.002). Moreover, based on HER2 receptor status, two patients received HER2 receptor targeting agent, trastuzumab deruxtecan (T-DXd), based on enrollment in clinical trials. One patient had stage 3 disease (squamous cell histology, PD-L1 CPS 5, TMB 11 mut/Mb, and HER2 2+) and was initially treated with CCRTx but showed disease progression in the pelvis, lung, and supraclavicular lymph nodes after treatment with second-line chemotherapy. This patient received T-DXd for 1 year and is still undergoing treatment. The other patient had stage 4 disease with ovarian metastasis (adenocarcinoma histology, PD-L1 CPS 5, TMB 6.3 mut/Mb, TP53 mutation, CCNE1 and ERBB2 amplification, and HER3 3+). After treatment with paclitaxel, carboplatin, and bevacizumab, this patient experienced recurrence in the vaginal vault and lung and received T-DXd as second-line therapy for 19 months, and is still undergoing treatment.

**Figure 3:**
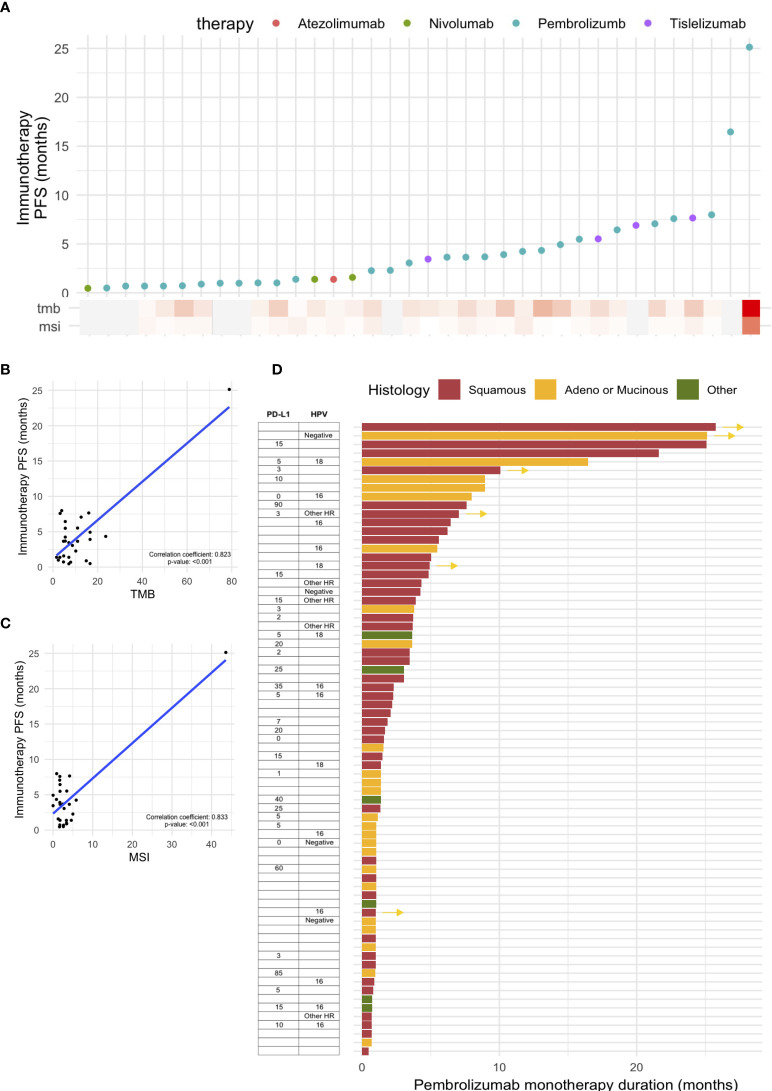
Outcomes of immunotherapy in cervical cancer. **(A)** Progression-free survival (PFS) based on various immunotherapy agents. PFS based on **(B)** tumor mutational burden (TMB) and **(C)** microsatellite instability (MSI). **(D)** Swimmer plot showing PFS based on pembrolizumab monotherapy, with PD-L1 CPS score and histology.

A total of 81 patients with cervical cancer underwent immunotherapy; monotherapy agents were: pembrolizumab (n = 73), tislelizumab (n = 4), nivolumab (n = 3), and atezolimumab (n = 1) (**Figure 3A**). Among the patients who underwent NGS, the median TMB was 6.3 mut/Mb (range 0.3–78.9) and the median MSI was 2.5% unstable sites (range 0–43.6). The progression-free survival (PFS) rate based on the type of immunotherapy with TMB and MSI values in these patients is shown in **Figure 3A**. Both TMB and MSI were significantly correlated with the duration of immunotherapy (**Figures 3B, C**). One patient with exceptionally high TMB and MSI in the scatter plots showed a durable response to pembrolizumab monotherapy; this patient had stage III adenocarcinoma and was negative for HPV. The presence of statistical significance in the correlation plots was dependent on the inclusion of this patient; if the patient was excluded, the significance was not observed (**Figure S2**).

For patients who received pembrolizumab monotherapy, a swimmer plot based on histology, PD-L1 CPS, and HPV status is shown in **Figure 3D**. The median age of patients receiving pembrolizumab was 48 (range: 32–80). In terms of ECOG status, 34.4% and 37.9% of the patients had ECOG statuses of 0 and 1, respectively; those with ECOG statuses 2 and 3 (17.2% and 10.3%, respectively) were also included in this retrospective study. In terms of prior lines of therapy, a majority (71.3%) had received one or two prior lines of treatment, but a sizable portion had also received three (17.2%) or four (10.3%). Most patients (23.3%) received pembrolizumab for one cycle as palliative care; five patients (6.8%) received pembrolizumab for ≥12 months, with two patients still undergoing treatment; and eight patients (11.0%) received pembrolizumab for 6–12 months, with two patients still undergoing treatment. The two patients with an exceptional response (≥12 months) to pembrolizumab monotherapy and who are still undergoing treatment both had advanced disease with lung metastasis, with or without brain metastasis. One patient who did not undergo NGS had exceptionally high PD-L1 SP263 (80%). Another patient showed a high mutational burden involving pathogenic SNV alteration of 15 genes and exhibited TMB of 78.9 mut/Mb and MSI 43.6% unstable MSI sites; PD-L1 was not tested.

## 4 Discussion

Cervical cancer diagnosed at an advanced-stage or in a recurrent disease setting is difficult to manage. Currently, many clinical trials on various immunotherapy agents and other targeted therapies, such as HER2 antibody–drug conjugates, are still ongoing to identify effective treatments for advanced or recurrent cervical cancer. Moreover, the identification of potential biomarkers using IHC and genomic assays is also important for personalized treatment for patients with advanced cervical cancer. The present study is a sizable study on patients with cervical cancer undergoing NGS with a well-described commercial panel. Most patients underwent TruSight Oncology 500 testing, which also provided the TMB and MSI values. We also included all patients with cervical cancer who underwent immunotherapy during the same period. This study provides real-world findings that encompass clinical, genomic, and treatment data, as well as outcomes of immunotherapy.

Previous studies have focused on either genomic alterations or therapeutic outcomes in cervical cancer. The landmark studies by the Cancer Genome Atlas (TCGA) (11, 15) have reported that the most frequent mutations in cervical cancer are PIK3CA (26%), EP300 (11%), FBXW7 (11%), and PTEN (8%) (11). In our study, PIK3CA mutations were identified in 23 of the 74 (31.1%) patients who underwent NGS; this rate is similar to or slightly higher than that in TCGA data, which are mostly based on Caucasian populations. In terms of prevalent mutations other than PIK3CA, our study revealed frequent mutations in TP53 (15 patients, 20.3%) and STK11 (6 patients, 8.1%), which is inconsistent with TCGA study. This difference may partially be attributed to the differences in histological subtypes, as approximately 75% of the cases were squamous cell histology in TCGA study, whereas only 52.8% of the patients had squamous cell histology in our cohort. Similar to our results, a previous study from China reported TP53 and STK11 mutations in 16% and 7% of the patients, respectively, suggesting ethnicity-related differences (16).

In the present study, we revealed that the genomic differences in SNVs and CNVs were partially associated with histology, as previously reported (15). We further investigated whether the HPV genotype may be associated with histology and found that among the most common subtypes, HPV 16 and HPV 18 may be associated with squamous cell type and adeno or mucinous type, respectively. Moreover, patients negative for HPV showed a similar distribution of histological subtypes to patients with HPV 16, suggesting the possibility of a false-negative HPV genotype in these patients. However, recent literature has suggested that HPV-independent cervical cancers may have different biology, bearing implications in carcinogenesis and treatment response (17). Additionally, owing to the advent of HPV vaccines, cervical cancers that originate from genotypes that are not covered by HPV vaccines or are HPV-independent may become more important in the future. Further studies on HPV based on NGS-based tests may help in further investigations (18).

The present study also highlights the potential use of IHC-based biomarkers for directing therapeutic options in cervical cancer. The PD-L1 CPS was particularly high in patients with squamous cell histology, with a median of 15. Patients with HPV 16 also showed a trend of high PD-L1 CPS, despite the lack of statistical significance owing to the limited number of patients with HPV genotype data. These findings suggest that patients with a high PD-L1 CPS may be candidates for immunotherapy, as suggested by previous clinical trials such as Keynote 158 and Checkmate 358 (6, 8, 9). Furthermore, our study showed that HER2 was not expressed in patients with squamous cell histology, whereas HER2 2+ and 3+ were identified in patients with non-squamous histology. Previous studies have indicated that a small, yet meaningful, proportion of patients with cervical cancer overexpress the HER2 receptor (19, 20). In our study, two patients overexpressing HER2 received T-DXd and showed a good response considering the treatment setting. The ongoing study, DESTINY-PanTumor02 (NCT04482309), will help to further investigate the effectiveness of T-DXd in the treatment of cervical cancer.

For immunotherapy outcomes, we found that the significant correlation between TMB and MSI with the duration of immunotherapy was largely driven by one patient with exceptionally high TMB and MSI showing a durable response. The calculation of the TMB and MSI using a panel-based sequencing approach may differ from the gold standard method of whole-exome sequencing-based testing and PCR-based testing of 5 MSI sites (21, 22). Although previous studies have suggested a high concordance between panel-based testing and the gold-standard methods (23), harmonizing data and establishing cutoffs across different panel designs remain challenging (24, 25).

In addition, the cohort characteristics are another, and perhaps more potent, confounder of immunotherapy outcomes. In the present study, our cohort represents real-world data based on the use of immunotherapy to treat cervical cancer. We revealed that the proportion of patients with a durable response is significantly lower than that observed in controlled clinical trial settings. A summary table comparing the present study with previous trials on mono-immunotherapy is given as **Supplementary Table 3** (6, 9, 26). Unlike prospective clinical trial settings where only patients with good performance scores (ECOG 0 or 1) are included, our retrospective cohort included heavily pre-treated patients, approximately 40% of whom showed poor performance scores. Furthermore, about one-fourth of the patients received only one cycle of pembrolizumab for palliative purposes, and for most of these patients, it was the last therapy attempted before death. These cohort characteristics, including the use of palliative treatment and limited testing for PD-L1 CPS, pose difficulty in interpreting biomarkers in our study, such as TMB, MRI, or PD-L1 CPS. Despite these limitations, we still observed that few patients showed a durable response, and these patients had high PD-L1 SP263 or high TMB/MSI, which could be predicted based on the known biomarkers for immunotherapy.

This study had some limitations. First, this study is a retrospective study; although we collected clinical variables, IHC, and NGS data, these data were selectively tested based on the clinicians’ discretion and may have caused potential selection bias. In addition, the choice of therapy was not based on systemic evaluation of a specific biomarker and was heterogeneous. As this was a single-center design, the practice patterns for IHC testing and immunotherapy use may differ in other centers. Moreover, the analysis was limited by the number of patients, especially because certain patients were not tested for certain biomarkers.

In conclusion, despite these limitations, our study represents a sizable cohort of patients with cervical cancer who underwent NGS with TruSight Oncology 500 or TruSight Tumor 170 panels, which are frequently used worldwide. To our knowledge, our study is the first to cover clinical variables, IHC results, genomic data, and immunotherapy outcomes. We found a considerable discrepancy between expected outcomes based on clinical trials and actual therapy outcomes in an unselected setting. These findings will help discuss therapeutic options with patients and identify new biomarkers or therapeutic agents for cervical cancer.

## Data availability statement

The raw data supporting the conclusions of this article will be made available by the authors, without undue reservation.

## Ethics statement

The studies involving human participants were reviewed and approved by Severance Hospital Institutional Review Board (IRB No # 4-2022-1399). Written informed consent for participation was not required for this study in accordance with the national legislation and the institutional requirements.

## Author contributions

Conceptualization: Y-NK and J-YL. Data curation: Y-NK and KL. Formal analysis: Y-NK and J-YL. Investigation: Y-NK, KL, EP, and J-YL. Methodology: Y-NK, EP, and J-YL. Supervision: JP, YL, J-YL, EN, SaK, SuK, and YK. Validation: EN, SaK, SuK, and YK. Visualization: Y-NK and J-YL. Writing – original draft: Y-NK. Writing – review and & editing: Y-NK, KL, EP, JP, YL, J-YL, EN, SaK, SuK, and YK. All authors contributed to the article and approved the submitted version.
