# Supplementary figures and images for: Comprehensive genomic and immunohistochemical profiles and outcomes of immunotherapy in patients with recurrent or advanced cervical cancer
Source: Front Oncol. 2023 May 15;13:1156973. doi: 10.3389/fonc.2023.1156973 (PMC10225637; doi:10.3389/fonc.2023.1156973)

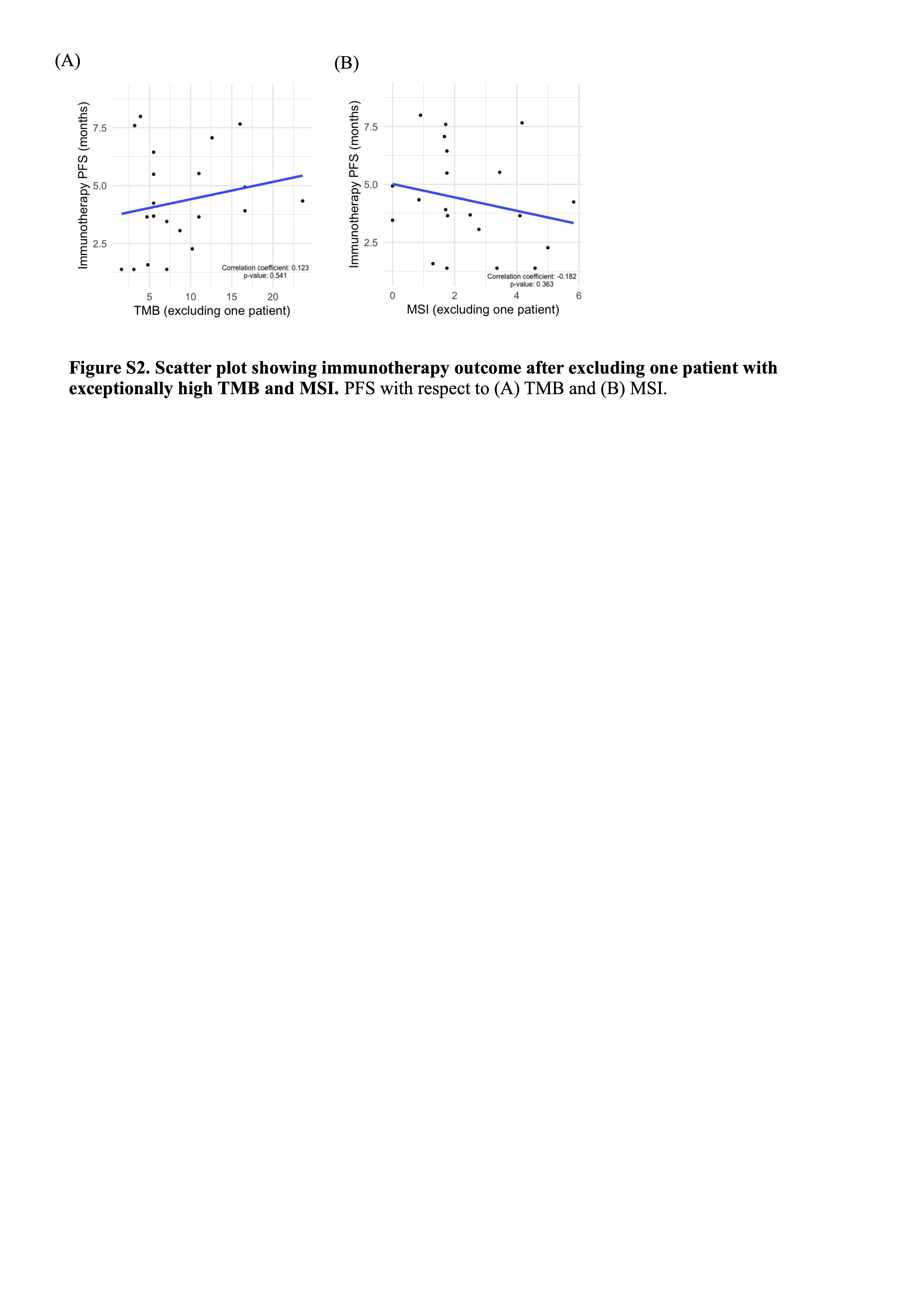

Supplement: Supplementary file 2 [file Image_2.jpeg]
