# Supplementary material for: Comprehensive genomic and immunohistochemical profiles and outcomes of immunotherapy in patients with recurrent or advanced cervical cancer
Source: Front Oncol. 2023 May 15;13:1156973. doi: 10.3389/fonc.2023.1156973 (PMC10225637; doi:10.3389/fonc.2023.1156973)
Supplement: Supplementary file 3 [file Table_1.docx]

| Variables | All patients (n=74) |
| --- | --- |
| Age (median, range) | 51 (32 – 80) |
| Detailed histology  Squamous cell carcinoma  Adenocarcinoma  Adenosquamous  Mucinous  Neuroendocrine  Clear cell  Serous  Carcinosarcoma | 39 (52.7%)  12 (16.2%)  4 (5.4%)  8 (10.8%)  8 (10.8%)  1 (1.4%)  1 (1.4%)  1 (1.4%) |
| FIGO stage  1A  1B  2A  2B  3B  3C  4  Recurrent | 2 (2.7%)  10 (13.5%)  4 (6.1%)  12 (13.6%)  4 (6.1%)  17 (24.2%)  17 (21.2%)  8 (12.1%) |
| Targeted therapy or immunotherapy  Pembrolizumab  Tislelizumab  Nivolumab  Atezolimumab  Herceptin | 21 (31.8%)  4 (6.1%)  3 (4.5%)  1 (1.5%)  2 (3.0%) |
|  |  |

**Table S1.** Patient characteristics
