# Supplementary material for: Comprehensive genomic and immunohistochemical profiles and outcomes of immunotherapy in patients with recurrent or advanced cervical cancer
Source: Front Oncol. 2023 May 15;13:1156973. doi: 10.3389/fonc.2023.1156973 (PMC10225637; doi:10.3389/fonc.2023.1156973)
Supplement: Supplementary file 4 [file Table_2.docx]

| Variables | Squamous cell  (n=39) | Adeno or Mucinous  (n=24) | Other  (n=11) | p-value |
| --- | --- | --- | --- | --- |
| HPV genotype (n=52)  16  18  Other high risk  Not detected | (n=26)  12 (46.2%)  1 (3.8%)  7 (26.9%)  6 (23.1%) | (n=19)  6 (31.6%)  7 (36.8%)  2 (10.5%)  4 (21.1%) | (n=7)  2 (28.6%)  3 (42.9%)  0 (0.0%)  2 (28.6%) | 0.049 |
| Tumor markers  SCC*  CA125**  CYFRA*** | 8.1 (0.4 – 70.0)  58.2 (13.0 – 99.7)  7.1 (7.1 – 7.1) | - 1. (0.4 – 11.0)   26.6 (4.5 – 4024.0)  3.8 (1.3 – 7.8) | 1.4 (0.7 – 9.4)  16.3 (7.2 – 582.8)  2.9 (1.2 – 4.6) | 0.019  0.799  0.537 |
| PD-L1 CPS**** | 15 (0 – 90) | 5 (0 – 5) | 5 (0 – 60) | 0.015 |
| HER2 expression  0  1  2  3 | (n=22)  20 (90.9%)  1 (4.5%)  1 (4.5%)  0 | (n=9)  3 (33.3%)  2 (22.2%)  4 (44.4%)  0 | (n=8)  5 (62.5%)  2 (25.0%)  0  1 (12.5%) | 0.002 |

* available in 53 patients (36 squamous cell, 10 adeno or mucinous, 7 other)

** available in 32 patients (3 squamous cell, 20 adeno or mucinous, 9 other)

*** available in 7 patients (1 squamous cell, 4 adeno or mucinous, 2 other)

**** available in 45 patients (22 squamous cell, 13 adeno or mucinous, 10 other)

**Table S2.** Clinical variables stratified by histological type
