# Supplementary material for: Comprehensive genomic and immunohistochemical profiles and outcomes of immunotherapy in patients with recurrent or advanced cervical cancer
Source: Front Oncol. 2023 May 15;13:1156973. doi: 10.3389/fonc.2023.1156973 (PMC10225637; doi:10.3389/fonc.2023.1156973)
Supplement: Supplementary file 5 [file Table_3.docx]

|  | Current study  (pembrolizumab monotherapy) | Keynote 158  (pembrolizumab) | Keynote 028  (pembrolizumab) | Checkmate 358  (nivolumab) |
| --- | --- | --- | --- | --- |
| Trial design | Retrospective | Phase II | Phase IB | Phase I/II |
| Cohort size (N) | 29 | 98 | 24 | 19 |
| Age (median, range) | 48 (32 – 80) | 46 (24 – 75) | 42 (26 – 62) | 51 (28 – 75) |
| Prior lines  1  2  3  4+ | 34.4%  37.9%  17.2%  10.3% | 30.6%  34.7%  16.3%  14.3% | 37.5%  25.0%  37.5% (three or more) | 42.1%  42.1%  15.8% |
| ECOG  0  1  2  3 | 19.5%  39.1%  10.3%  31.0% | 34.7%  65.3% | 25.0%  75.0% | 58%  42% |
| PD-L1 | 50% (among tested) ^*^ | 83.7%^*^ | 100%^#^ | 52.6%^%^ |
| BOR  CR  PR  SD  PD  Not assessed | 0  2 (6.9%)  10 (34.5%)  16 (55.1%)  1 (3.4%) | 3 (3.1%)  9 (9.2%)  18 (18.4%)  55 (56.1%)  13 (13%) | 0  4 (16.7%)  3 (12.5%)  16 (66.6%)  1 (4.2%) | 3 (16%)  2 (10%)  8 (42%)  6 (32%)  0 |

ECOG, Eastern Cooperative Oncology Group; PD-L1, programmed death-ligand 1; BOR, best overall response; CR, complete response; PR, partial response; SD, stable disease; PD, progressive disease. ^*^ ≥1 of combined positive score (CPS), ^#^≥1% of modified proportion score, ^%^≥1% of tumor proportion score

**Supplementary Table 3.** Comparison between current study and previous published trials on immunotherapy monotherapy.
